# Greenhouse Gas Emissions and Stem Aerenchyma Development of Rice Under Different Water and Nitrogen Regimes

**DOI:** 10.3390/biology15171514

**Published:** 2026-09-03

**Authors:** Sitthikorn Bodeerath, Chanakan Prom-u-thai

**Affiliations:** 1 Agronomy Division, Department of Plant and Soil Sciences, Faculty of Agriculture, Chiang Mai University, Chiang Mai 50200, Thailand; sitthikorn_bodi@cmu.ac.th; 2 Lanna Rice Research Center, Chiang Mai University, Chiang Mai 50100, Thailand

**Keywords:** sustainable rice cultivation, global warming, greenhouse gas emissions, greenhouse gas mitigation, aerenchyma tissue, rice stem

## Abstract

Rice cultivation is an important source of GHG emissions, particularly CH_4_ and N_2_O, which are strongly influenced by water and N management. However, the relationship between GHG emissions and stem aerenchyma development among rice varieties remains poorly understood. This study evaluated CH_4_ and N_2_O emissions and stem aerenchyma development in two rice varieties, PTT1 and KDML105, cultivated under flooded and non-flooded conditions with two N regimes (0 and 120 kg ha^−1^). GHG fluxes were measured at three growth stages: before maximum tillering, panicle initiation, and flowering, while stem aerenchyma was assessed at the early heading stage. The results showed that water conditions and N fertilizer significantly affected cumulative CH_4_ emissions, whereas there was no significant effect of rice variety. Applying both water and N also increased GWP and GHGI in rice cultivation, with little difference between the rice varieties. Notably, stem aerenchyma development was not significantly associated with GHG transport under different conditions. These findings demonstrate that water and N management affected rice physiological responses, but stem aerenchyma development alone may not be associated with GHG transport under specific environmental conditions. These results provide valuable guidance for optimizing water and N management in rice production systems to maintain crop productivity while reducing environmental impacts.

## Simple Summary

Rice cultivation contributes to greenhouse gas (GHG) emissions and climate change. In this study, we compared the effects of contrasting water and nitrogen (N) regimes on the growth of two rice varieties, Pathum Thani 1 (PTT1) and Khao Dawk Mali 105 (KDML105). PTT1 produced a higher grain yield than KDML105. However, cumulative methane (CH_4_) emissions did not differ significantly between the two rice varieties. Under flooded conditions, N fertilization increased stem aerenchyma formation in both rice varieties, particularly KDML105. However, this response was not significantly associated with CH_4_ or N_2_O emissions. These findings suggest that water and N management affected the physiological responses of the rice varieties, which may influence both crop productivity and environmental impacts. Appropriate management could, therefore, contribute to more sustainable rice production.

## 1. Introduction

Greenhouse gases (GHGs) remain the primary drivers of climate change and are currently at record-high atmospheric concentrations, particularly carbon dioxide (CO_2_), methane (CH_4_), and nitrous oxide (N_2_O), largely due to anthropogenic activities [1]. Previous studies have reported that total global GHG emissions reached approximately 372,716.86 Gg CO_2_ eq, with the agricultural sector contributing about 22% of total emissions in Thailand, ranking third after the industrial and energy sectors [2,3]. Rice cultivation contributes approximately 9–11% of agricultural emissions, primarily through CH_4_, N_2_O, and CO_2_ emissions. A significant portion of rice cultivation requires water retention throughout the growing period, thereby creating an anaerobic environment conducive to methane-producing microorganisms [4,5]. However, several factors influence GHG emissions from rice systems, including rice variety, fertilizer utilization, water conditions, soil properties, and microorganisms in the soil [6].

Nitrogen (N) is a macronutrient that is crucial for plant growth and development. High doses of N fertilizer can not only be toxic to plants, resulting in decreased yield, but also enhance GHG emissions and exacerbate environmental impacts [7,8]. Globally, N fertilizer accounts for 58% of total fertilizer consumption [9]. Rice is recognized as a crop with relatively low nitrogen use efficiency (NUE) [10], with plants typically utilizing only 20–50% of applied N [11]. Consequently, inefficient N use contributes to environmental losses and increased GHG emissions. However, certain rice varieties (the so-called N-efficient varieties) exhibit improved NUE due to favorable morpho-physiological traits, including enhanced N uptake and remobilization, more extensive root systems, and higher photosynthetic NUE [12]. Optimizing N fertilizer management in rice production can increase rice yield and thus income while simultaneously reducing overall GHG emissions and carbon footprint [5].

It is well documented that continuous flooding is a widely used agricultural practice in global rice cultivation, except for upland rice or aerobic rice cultivation. This shifts the rhizosphere from anoxic to hypoxic conditions, posing a major constraint on plant growth and triggering complex physiological and genetic adaptations in rice [13]. A key adaptation in wetland plants is the formation of longitudinal aerenchyma channels that facilitate the diffusion of oxygen (O_2_) from the shoots to the root tips, while also serving as a major conduit GHG into the atmosphere, particularly through the leaf sheath, which is closely linked to the ethylene (C_2_H_4_) hormone signaling [14] and is strongly affected by N availability and water conditions [15,16]. Management conditions affect the activity of precursors in this pathway, such as 1-aminocyclopropane-1-carboxylic acid (ACC) synthase (ACS) and oxidase (ACO) [17], driven by the accumulation of reactive oxygen species (ROS), including superoxide anions and hydrogen peroxide [16]. However, most studies have focused on root aerenchyma tissues, while limited information is available on stem aerenchyma, particularly regarding water and N fertilizer regimes, which are common practices in rice cultivation. The dynamics of N and aeration and their effects on morpho-physiological characteristics, including shoot and root biomass and tissue N concentration, are influenced by this interaction [18]. However, the effects of combined water and N management on stem aerenchyma development and the resulting GHG fluxes and cumulative emissions have not been examined in rice varieties.

Therefore, the objectives of this study were to (1) evaluate GHG emissions from rice varieties grown under contrasting water and N conditions and (2) determine whether different water and N conditions influence stem aerenchyma formation and GHG emissions in rice varieties. We hypothesized that stem aerenchyma development and GHG emissions respond differently to contrasting water and N regimes in rice cultivation. The findings provide crucial physiological insights for environmental management that may help explain differences in N utilization and gas exchange capacities in rice under varying cultivation practices. Appropriate management of environmental practices is a key strategy for reducing economic costs and total GHG emissions in rice cultivation.

## 2. Materials and Methods

### 2.1. Plant Material and Growing Conditions

A pot experiment was arranged in a factorial randomized complete block design (RCBD) with three independent replications comprising the N regime, water management, and rice variety. Water management under flooded and non-flooded conditions was applied in this experiment, with two N fertilizer treatments: non-N application (basal fertilizer; control treatment) and soil N application (N fertilizer at 120 kg N ha^−1^; optimum N rate for rice in Thailand [19,20]). The two rice varieties used in this study were Pathum Thani 1, PTT1 (modern improved high-yielding variety, insensitive to photoperiodism) and Khao Dawk Mali 105, KDML105 (traditional popular jasmine rice variety, sensitive to photoperiodism). The plants were grown during the rainy season (July to November) in 2025 at the research station of Chiang Mai University (18°47′ N, 98°57′ E). The average temperature during the cropping season was 27.9 °C, with a relative humidity of 80.2%. The average precipitation during grain filling was 2.46 mm [21]. The soil in the experimental treatments had a sandy loam soil texture, with pH 4.59 (1:1, soil/water), 5.13% organic matter (Walkley–Black method), 0.07% total N (combustion method), 553.76 mg kg^−1^ available phosphorus (Bray II), and 254.10 mg kg^−1^ exchangeable potassium (NH_4_OAc pH7/AES) with a bulk density of 1.36 g cm^−3^. For seedlings, the seeds were surface-sterilized with 70% ethanol and 1% NaOCl for five minutes and then rinsed three times with deionized water. The rice plants were grown from seeds to seedlings in a seedling tray (55 × 36 × 4 cm) over 21 days. The two seedlings per variety were transplanted into plastic pots (radius 12.0 cm and height 21.0 cm), which contained the soil in an amount of ~10 kg DW pot^−1^. Basal fertilizer containing P and K at 75 kg P_2_O_5_ ha^−1^ and 75 kg K_2_O ha^−1^, respectively, was applied a day before transplanting. N fertilizer in the form of urea (46-0-0) was applied two times in an amount of 0.59 and 0.59 g pot^−1^ 3 days before GHG measurement at the tillering and panicle initiation stages. The pot was permanently flooded with five cm of water through plant maturity, maintaining the plants under flooded conditions. The soil moisture levels were maintained at field capacity between 20 and 30% [22], and soil moisture was detected using a soil moisture meter (HH2 Moisture Meter with ML2x Theta Probe, Delta-T Devices Ltd., Cambridge, UK) under non-flooded conditions. Weeds were removed manually.

### 2.2. Sample Collection and N Analysis

Samples were harvested from each pot at the maturity stage to assess the grain yield, straw dry weight, and the various yield components, including plant height, the number of tillers per plant, the number of panicles per plant, the number of spikes per panicle, the percentage of filled grains, and the 1000-grain weight. The grain yield was measured at a moisture content of 14%, and the straw dry weight was determined after oven drying at 75 °C for 72 h. The harvest index was calculated as the grain yield divided by the total biological yield.

The collected samples comprising the shoot and grains were oven-dried at 75 °C for 72 h and ground for 20 s using a hammer mill (Scientific Technical Supplies D-6072, Dreieich, Germany). N concentration was measured by combustion analysis utilizing a LECO combustion furnace (LECO FP-828, Saint Joseph, MI, USA). After being weighed into a foil cup, the samples were burned in an oxygen-flowing, high-temperature chamber to produce CO_2_, SO_2_, N_2_, and NO_x_ from elemental carbon, N, and sulfur. The gases were examined using infrared absorption and thermal conductivity detection to determine the proportion of N in terms of percentage (%). Then, the N concentration was converted to g kg^−1^. The N content of each plant part was calculated by multiplying the plant part’s dry weight by the N concentration in each part. The total uptake of N was calculated as the summation of the N contents in all plant parts. The NUE parameters, namely, recovery nitrogen use efficiency (RNUE, %), physiological nitrogen use efficiency (PNUE, g g^−1^), and agronomic nitrogen use efficiency (ANUE, g g^−1^), were calculated as described in Fageria et al. [23].

### 2.3. Greenhouse Gas Flux, Cumulative Global Warming Potential, and Emissions Intensity

CH_4_ and N_2_O fluxes were measured using a static closed chamber method. The chamber measured 30 × 30 cm (0.09 m^2^) and was equipped with an internal circulation fan to ensure homogeneous gas mixing during sampling. All gas sampling was performed between 09:00 and 11:00 AM and 02.00 and 05.00 PM during three critical growth stages: 26 days after transplanting (before maximum tillering), at panicle initiation (46 days after transplanting), and at flowering (74 and 80 days after transplanting in PTT1 and KDML105, respectively) using water as an air barrier instead of the chamber base. The purpose is to analyze trends in gas emissions during this critical growth period. At each sampling point, 30 mL of gas was withdrawn from the chamber headspace using a 35 mL airtight syringe fitted with a three-way stopcock at 0, 5, and 15 min following chamber closure. Each sample was immediately transferred into a 20 mL pre-evacuated glass vial and sealed for laboratory analysis. The chamber air temperature was simultaneously recorded at each time interval using an alcohol thermometer. CH_4_ and N_2_O concentrations were determined by gas chromatography (GC) using a flame ionization detector (FID) and an electron capture detector (ECD), respectively. The specific operation conditions for the GC are described in Cha-un et al. [24]. Cumulative CH_4_ and NO_2_ fluxes were calculated by integrating flux measurements collected 26 days after transplanting through to harvest, covering the panicle initiation and flowering stages.

CH_4_ and N_2_O emissions were expressed as global warming potential (GWP) on a CO_2_-equivalent basis. CH_4_ and N_2_O emissions were converted using GWP conversion factors based on a 100-year time horizon in accordance with the Sixth Assessment Report (AR6) of the Intergovernmental Panel on Climate Change (IPCC). GWP conversion factors of 27 and 273 were applied for CH_4_ and N_2_O, respectively. Then, greenhouse gas emission intensity (GHGI) was estimated by dividing GWP by the grain yield, given in the units of g g^−1^ according to the methods described in Fawibe et al. [25] and Yang et al. [26], respectively.

### 2.4. Aerenchyma Characteristics

Shoot aerenchyma was measured using three independent stems per plant that had completely headed (14-week-old) for each section [18]. The number of internal nodes was counted by observation. Internodal sections between 0 and 10, 10 and 20, and 20 and 30 mm above the coleoptilar and the first and second nodes of each treatment were observed. Cross-sections (1.0 mm thick) of the long internodes were stained with Lugol’s solution (1:2 I/KI) for 60 s and rinsed three times with water before visualizing starch to detect pre- and aerenchyma cells (these cells contain very little starch compared to parenchyma cells) using a stereomicroscope (Zeiss Stemi 508, Carl Zeiss Microscopy Deutschland GmbH, Oberkochen, Germany) at 40× magnification.

The characteristics of aerenchyma tissue (cell number and percentage of aerenchyma tissue) were determined at the early heading stage. Aerenchyma tissue characteristics were calculated from the section images using ImageJ v. 1.54g software (National Institutes of Health, Bethesda, MD, USA). The number of aerenchyma cells was counted, and stem radius (cm) was determined from the section images. Total cell area was calculated by summing the number of aerenchyma cells. The stem aerenchyma formation was calculated by dividing the area of aerenchyma cells by the total area of the stem and expressed as a percentage. The stem samples from each treatment among varieties were oven-dried at 75 °C for 72 h. Then, the dry weight of the stem was measured, and the stem was ground for 20 s using a hammer mill (Scientific Technical Supplies D-6072, Dreieich, Germany) for N analysis.

### 2.5. Statistical Analysis

The data are presented as mean ± standard errors (SEs) for each trait and treatment; water conditions (W), rice varieties (V), and N regimes (N) are based on three independent biological replicates within each treatment. Statistical analyses were performed using Statistix v10 software (Analytical Software, Tallahassee, FL, USA). Differences among traits were analyzed using three-way analysis of variance (ANOVA) followed by using least significant difference (LSD) tests. The homogeneity of variance and normality were examined using the Shapiro–Wilk test and Levene’s test. Statistical significance was defined as *p* < 0.05. Pearson correlation analysis was used to examine the relationship between variables. All figures were produced using MS Excel, and bars show the SEs.

## 3. Results

### 3.1. Yield and Yield Components

**Figure 1:**
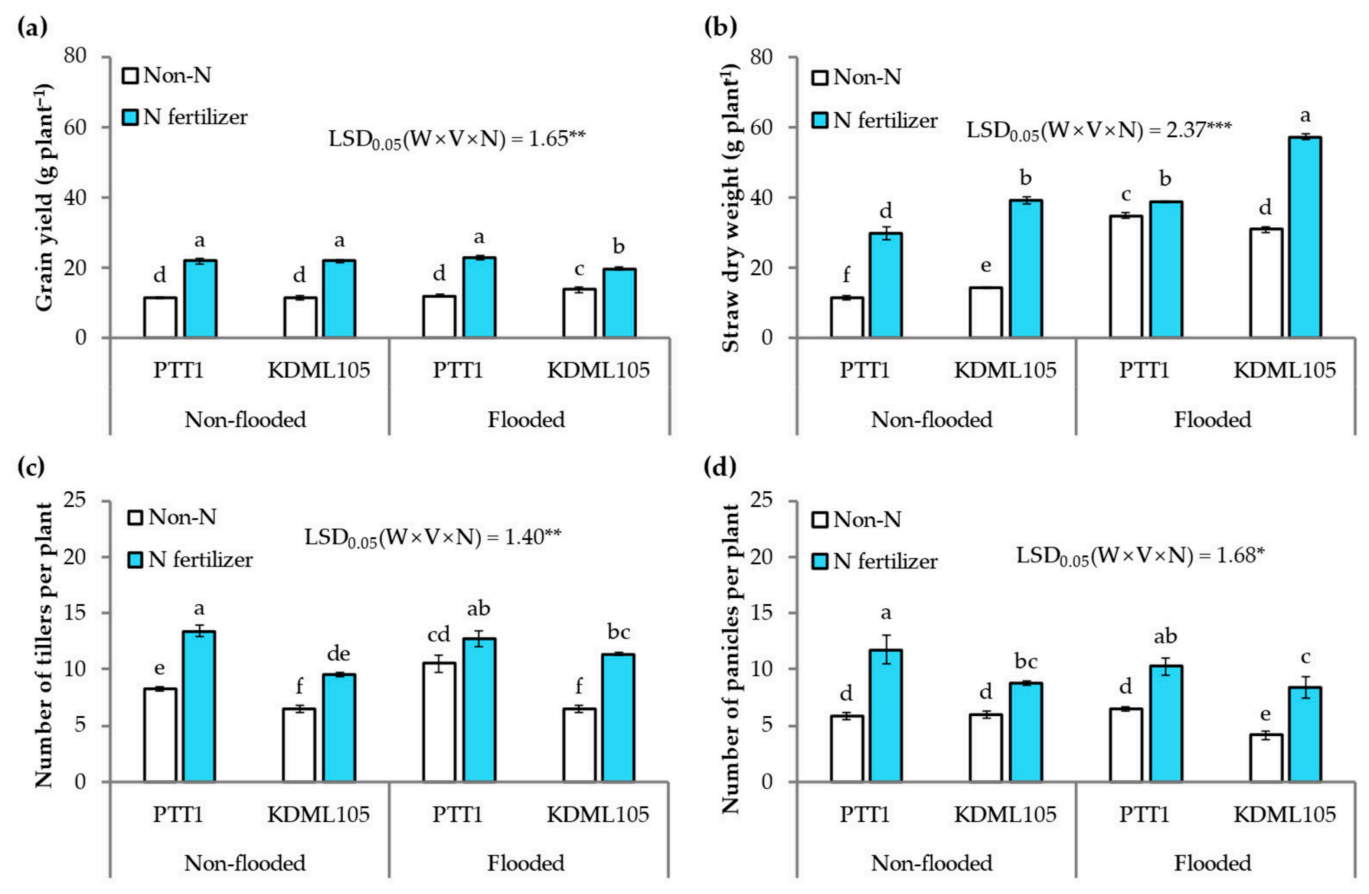
Yield and yield components between PTT1 and KDML105 rice varieties. The grain yield (**a**), straw dry weight (**b**), tiller (**c**), and panicle number per plant (**d**). Values represent the mean ± SE of three biological replicates. Error bars are SEs, and different letters indicate significant differences between water conditions (W), rice varieties (V), and nitrogen regimes (N), while the asterisks indicate significant differences between variables by LSD at * *p* < 0.05, ** *p* < 0.01, and *** *p* < 0.001.

Grain yield was affected by the interaction effect between water conditions and N fertilizer application (Figure 1a). Under non-flooded conditions, the control produced similar grain yield (average, 11.42 g plant^−1^) in both varieties. Application of N fertilizer significantly increased grain yield by 2.0-fold compared to their respective controls. Under flooded conditions, PTT1 produced a higher grain yield than KDML105 when N fertilizer was applied, but the opposite was observed when N fertilizer was not applied. Applying N increased grain yield in both varieties to different degrees, with the highest increase in PTT1. Straw dry weight showed a similar interaction of water conditions and N fertilizer application as in grain yield (Figure 1b). Under non-flooded conditions, the control treatment produced 11.6 and 14.4 g plant^−1^ in PTT1 and KDML105, respectively. Applying N fertilizer increased straw dry weight by 2.6- and 2.7-fold compared to the controls, respectively. The straw dry weight increased in both varieties under flooded conditions and N application. In KDML105, the straw dry weight increased by 1.9-fold relative to the control (30.1 g plant^−1^), the highest among the varieties and N treatments. Similar interaction effects were observed in yield components, especially the numbers of tillers and panicles per plant (Figure 1c,d). The variety PTT1 exhibited more tillers and panicles than KDML105 when N fertilizer was applied under both non-flooded and flooded conditions.

### 3.2. Total Shoot N Content and Nitrogen Use Efficiency

**Figure 2:**
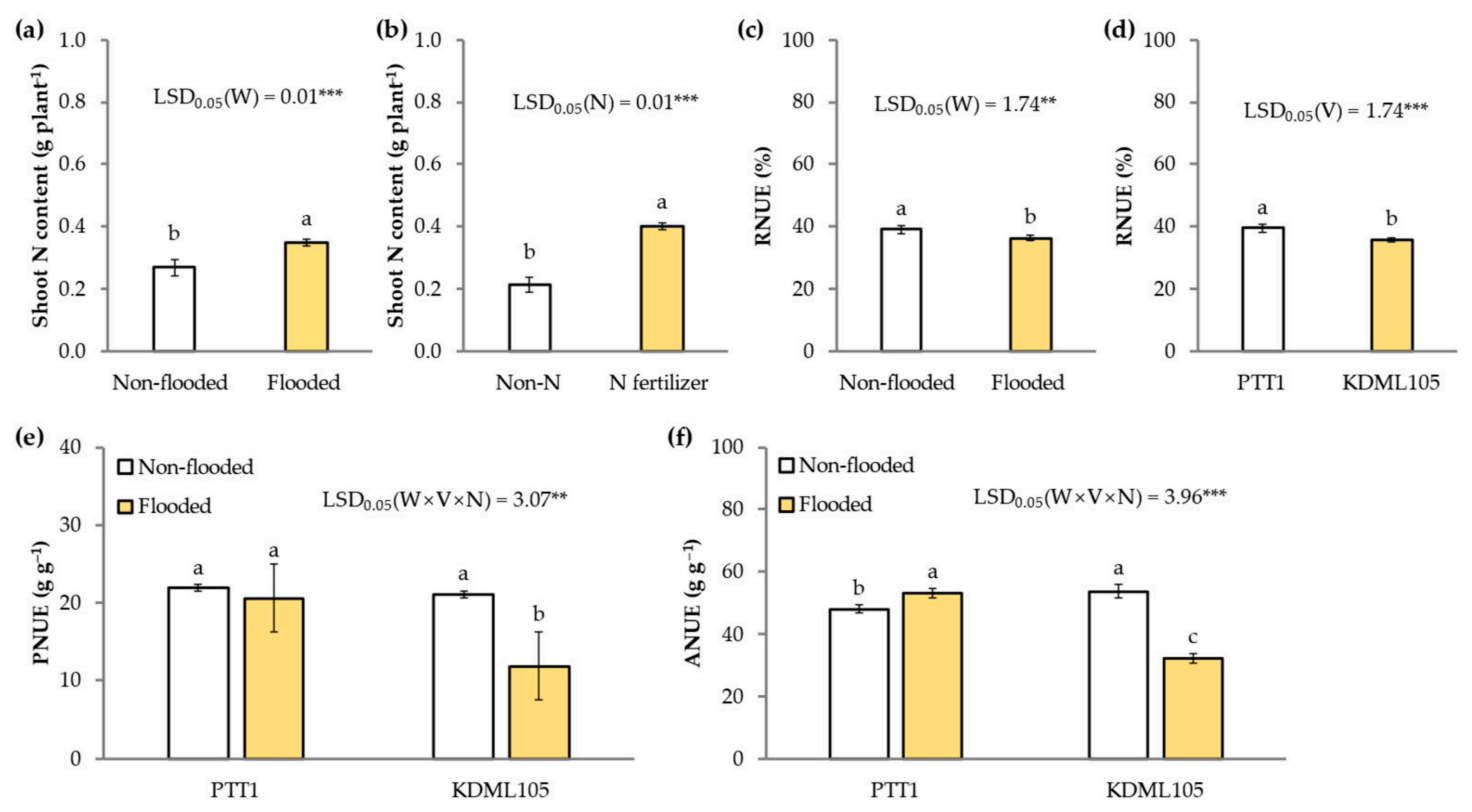
Total shoot N content and NUE traits between PTT1 and KDML105 rice varieties. The total shoot N content under different water conditions (**a**) and N regimes (**b**), NUE traits; recovery nitrogen use efficiency (RNUE) under different water conditions (**c**) and rice varieties (**d**), physiological nitrogen use efficiency (PNUE) (**e**), and agronomical nitrogen use efficiency (ANUE) (**f**). Values represent the mean ± SE of three biological replicates. Error bars are SEs, and different letters indicate significant differences between water conditions (W), rice varieties (V), and nitrogen regimes (N), while the asterisks indicate significant differences between variables by LSD at ** *p* < 0.01 and *** *p* < 0.001.

The water conditions and N fertilizer application affected the total shoot N content differently between the rice varieties, but there was no significant interaction (*p* < 0.05) (Figure 2a,b). Under flooded conditions, the rice plants accumulated N from the soil (average 0.35 g plant^−1^), which was higher than under non-flooded conditions. Applying N fertilizer increased N accumulation 1.9-fold compared to non-N application (0.21 g plant^−1^). In addition, there was a significant interaction between water conditions and N fertilizer applications on RNUE in both rice varieties, but there was no interaction between the two factors among N regimes (Figure 2c,d). Under non-flooded conditions, 39.1% of N was recovered from the plants, which was greater than under flooded conditions (36.3%). Moreover, PTT1 had a higher N recovery percentage (39.6%) than KDML105 (35.8%). There was a significant interaction between the factors for PNUE and ANUE (Figure 2e,f). PNUE and ANUE in KDML105 were decreased when cultivated under flooded conditions compared to non-flooded conditions, declining from 21.4 g g^−1^ and 55.6 g g^−1^ to 15.4 g g^−1^ and 39.1 g g^−1^, respectively. PTT1 cultivated under flooded conditions showed increased translocation of N to the yield (ANUE) by 26.5 g g^−1^, which was 1.2-fold higher than under non-flooded conditions. However, there was no significant effect on PNUE.

### 3.3. Greenhouse Gas Flux and Cumulative Emissions

#### 3.3.1. CH_4_ and N_2_O Flux Emissions

**Figure 3:**
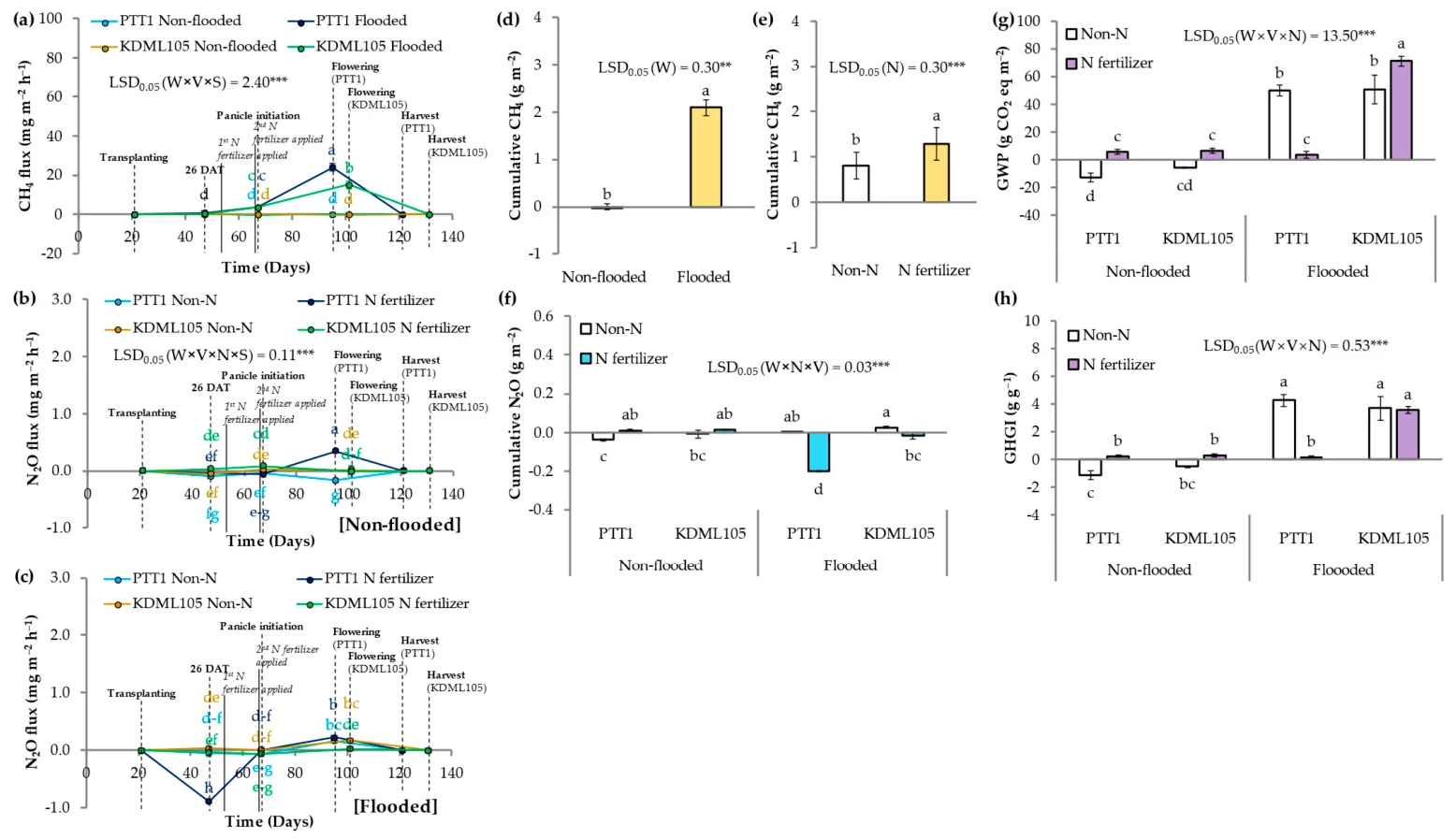
Greenhouse gas flux and cumulative emissions between PTT1 and KDML105 rice varieties. CH_4_ emissions (**a**) and N_2_O emissions at different growth stages under non-flooded (**b**) and flooded (**c**) conditions. Cumulative CH_4_ emissions under different water conditions (**d**) and nitrogen regimes (**e**). Cumulative N_2_O emissions (**f**). Global warming potential (GWP) (**g**) and greenhouse gas emissions intensity (GHGI) (**h**). For flux measurement, 21-day-old rice was transplanted into a pot experiment, and gas emissions were collected before maximum tillering (26 days after transplanting), panicle initiation (46 days after transplanting), and flowering stages (74 and 80 days after transplanting in PTT1 and KDML105, respectively). N fertilizer was applied 3 days before GHG measurement of two different growth stages at maximum tillering and panicle initiation. Values represent the mean ± SE of three biological replicates. Error bars indicate SEs, and different letters indicate significant differences between water conditions (W), rice varieties (V), and nitrogen regimes (N), while the asterisks indicate significant differences between variables by LSD at ** *p* < 0.01, and *** *p* < 0.001.

Most of the CH_4_ was emitted from the flooded soil. There was no significant interaction between water conditions and N fertilizer applications on CH_4_ cumulative emissions among rice varieties at the different growth stages (Figure 3a). There was a significant interaction on CH_4_ flux between water conditions and rice varieties at different growth stages, particularly at the flowering stage. PTT1 showed the highest CH_4_ flux (24.0 mg m^−2^ h^−1^), greater than KDML105 (15.1 mg m^−2^ h^−1^) when cultivated under flooded conditions, while the same did not occur under non-flooded conditions. N_2_O flux was differently affected by the interaction effect between water conditions and N fertilizer applications among rice varieties and growth stages (Figure 3b,c). The emissions were also elevated in the flowering stage of PTT1 with N fertilizer under non-flooded conditions, by 0.34 mg m^−2^ h^−1^, while PTT1 with N fertilizer had a flux that peaked at 0.22 mg m^−2^ h^−1^, followed by PTT1 and KDML105 without N application, with the same value of 0.17 mg m^−2^ h^−1^ under flooded conditions.

#### 3.3.2. Cumulative CH_4_ and N_2_O Emissions

There was no significant interaction between water conditions and N fertilizer applications on cumulative CH_4_ emissions among rice varieties, but water conditions and N fertilizer applications affected cumulative CH_4_ emissions (*p* < 0.05) (Figure 3d,e). Flooding produced cumulative CH_4_ of 2.1 g m^−2^ season^−1^, which was higher than non-flooding (0.06 g m^−2^ season^−1^). Similarly, N fertilizer application resulted in cumulative CH_4_ emissions of 0.8 g m^−2^ season^−1^ under non-N and 1.3 g m^−2^ season^−1^ under N fertilizer application. There was a significant interaction between the two factors in cumulative N_2_O emissions among rice varieties (Figure 3f). There were differences in cumulative N_2_O between rice varieties. The cumulative amount of N_2_O absorbed by PTT1 was 0.20 g m^−2^ season^−1^ with N under flooded conditions, and this declined to 0.03 g m^−2^ season^−1^ in KDML105 without N under flooded conditions.

#### 3.3.3. Global Warming Potential (GWP) and Greenhouse Gas Emissions Intensity (GHGI)

Our measures of GWP and GHGI followed from two components, namely, CH_4_ and N_2_O emissions, which were expressed as g CO_2_ eq m^−2^ to quantify the GWP among treatments. There was a significant interaction effect between water conditions and N fertilizer application on GWP and GHGI among rice varieties (Figure 3g,h). Interestingly, PTT1 had a lower effect on GWP and GHGI than KDML105, particularly in flooded conditions. Under non-flooded conditions, both rice varieties showed similar GWP and GHGI (6.34 g CO_2_ eq m^−2^ and 0.29 g g^−1^) values compared to those without N applied (−9.05 g CO_2_ eq m^−2^ and −0.81 g g^−1^). In contrast, PTT1 decreased GWP and GHGI (3.86 g CO_2_ eq m^−2^ and 0.17 g g^−1^, respectively) compared to the control (50.28 g CO_2_ eq m^−2^ and 4.29 g g^−1^, respectively). KDML105 also had a high GWP (50.58 g CO_2_ eq m^−2^) and a similarly high GHGI (3.72 g g^−1^) compared to without N applied under flooded conditions.

### 3.4. Aerenchyma Characteristics

**Figure 4:**
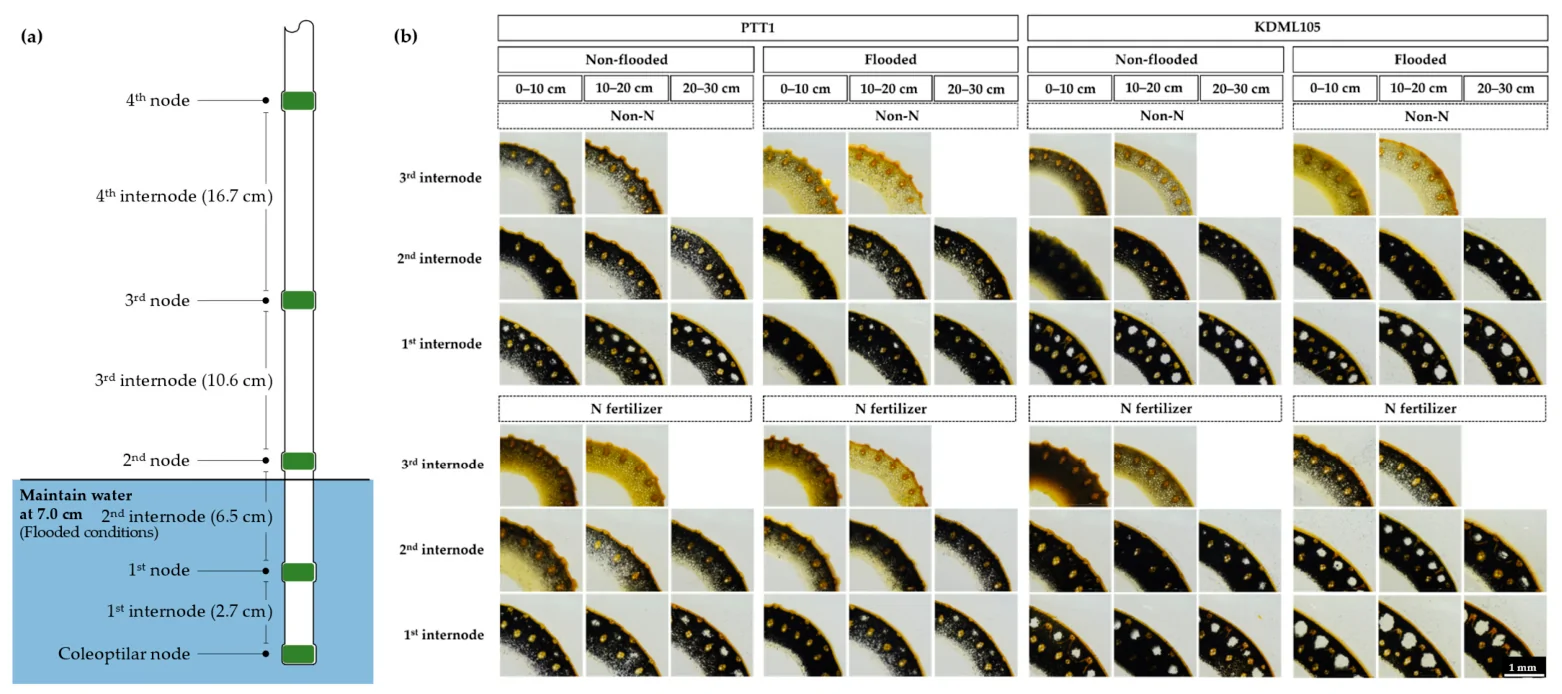
Developmental regulation of stem aerenchyma formation between PTT1 and KDML105 rice varieties. Each rice variety was partially submerged for 77 days after transplanting, with 7 cm under water (flooded conditions) or no submergence (non-flooded conditions) (**a**). The percentage of aerenchyma formation at 10, 20, and 30 mm above the coleoptilar and the first and second nodes was determined by staining for starch accumulation with Lugol’s solution. Cross-section of PTT1 and KDML105 under contrasting water and nitrogen regimes at the coleoptilar and the first and second nodes reveals an interstitial space within the parenchymatous cells, indicative of the presence of aerenchyma tissue (**b**). All images are representative of *n* = 3 replicates. Histological sections of rice stem at 40× magnification (Lugol’s solution staining, 1 mm scale bar).

Cross-sections of the first three nodes of the stems in the two rice varieties grown under different water and N fertilizer regimes were taken at three distances (0–10, 10–20, and 20–30 mm) (Figure 4a,b). After staining with Lugol’s solution, most rice varieties clearly developed aerenchyma tissue in the coleoptilar node, particularly at 10–20 mm and 20–30 mm from the node, with variation depending on water and N fertilizer conditions. Aerenchyma formation was observed in the first node of KDML105, whereas PTT1 showed limited aerenchyma development. In contrast, aerenchyma tissue was not detected in the second node at any measured distance in either rice variety. The stem morphology (number of nodes and aerenchyma, and the stem radius) was not significantly different between the PTT1 and KDML105 rice varieties grown under contrasting water and N regimes (Table 1).

**Figure 5:**
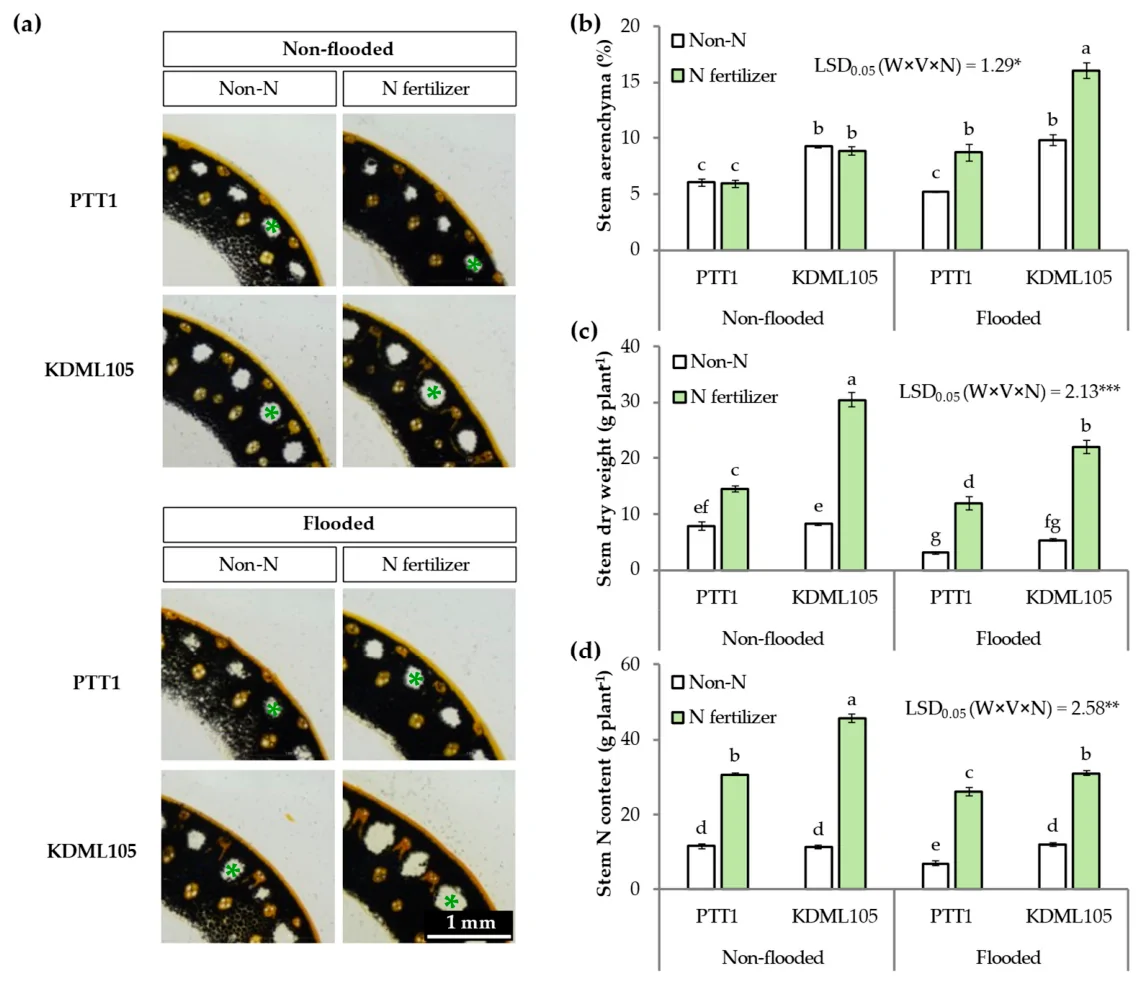
The sections of rice stems at the early flowering stage (77 days after transplanting) were excised at 20 mm above the coleoptilar node in PTT1 and KDML105 rice varieties and then stained for starch accumulation with Lugol’s solution. The responses of stem aerenchyma under contrasting water and N conditions (**a**), stem aerenchyma formation (**b**), stem dry weight (**c**), and stem N content (**d**). The percentage of stem aerenchyma formation was calculated using ImageJ software. Values represent the mean ± SE from three stems analyzed per treatment in three biological replicates. Error bars indicate SEs, and different letters indicate significant differences between water conditions (W), rice varieties (V), and N regimes (N), while the asterisks indicate significant differences between variables by LSD at * *p* < 0.05, ** *p* < 0.01, and *** *p* < 0.001. All images and data are representative of *n* = 3 replicates. Aerenchyma tissues are marked with a green asterisk. Histological sections of rice stem at 40× magnification (Lugol’s solution staining, 1 mm scale bar).

In contrast, there was a significant interaction between rice variety and N fertilizer application for stem aerenchyma formation under different growing conditions (Figure 5a,b). Under flooded conditions, the rice varieties showed different alterations in the percentage of aerenchyma formed under different N regimes. Applying N fertilizer increased the average percentage formation of aerenchyma by 1.64 times in both PTT1 and KDML105 compared with non-N, while such an effect was not observed under non-flooded conditions. Additionally, a significant difference in stem dry weight and N content under water and N conditions was observed between rice varieties (*p* < 0.05) (Figure 5c,d). Under non-flooded conditions, the stem dry weight and N content increased when N fertilizer was applied to PTT1 (by 3.9 and 3.8 times) and KDML105 (by 4.1 and 2.6 times) compared to non-N. The stem dry weight and N content increased when N fertilizer was applied to PTT1 (by 1.8- and 2.7-fold) and KDML105 (by 3.7- and 4.1-fold) compared to non-N under flooded conditions.

### 3.5. The Relationship Between Yield, Yield Components, Stem Morphological and Physiological Characteristics, and Cumulative Gas Emissions

**Figure 6:**
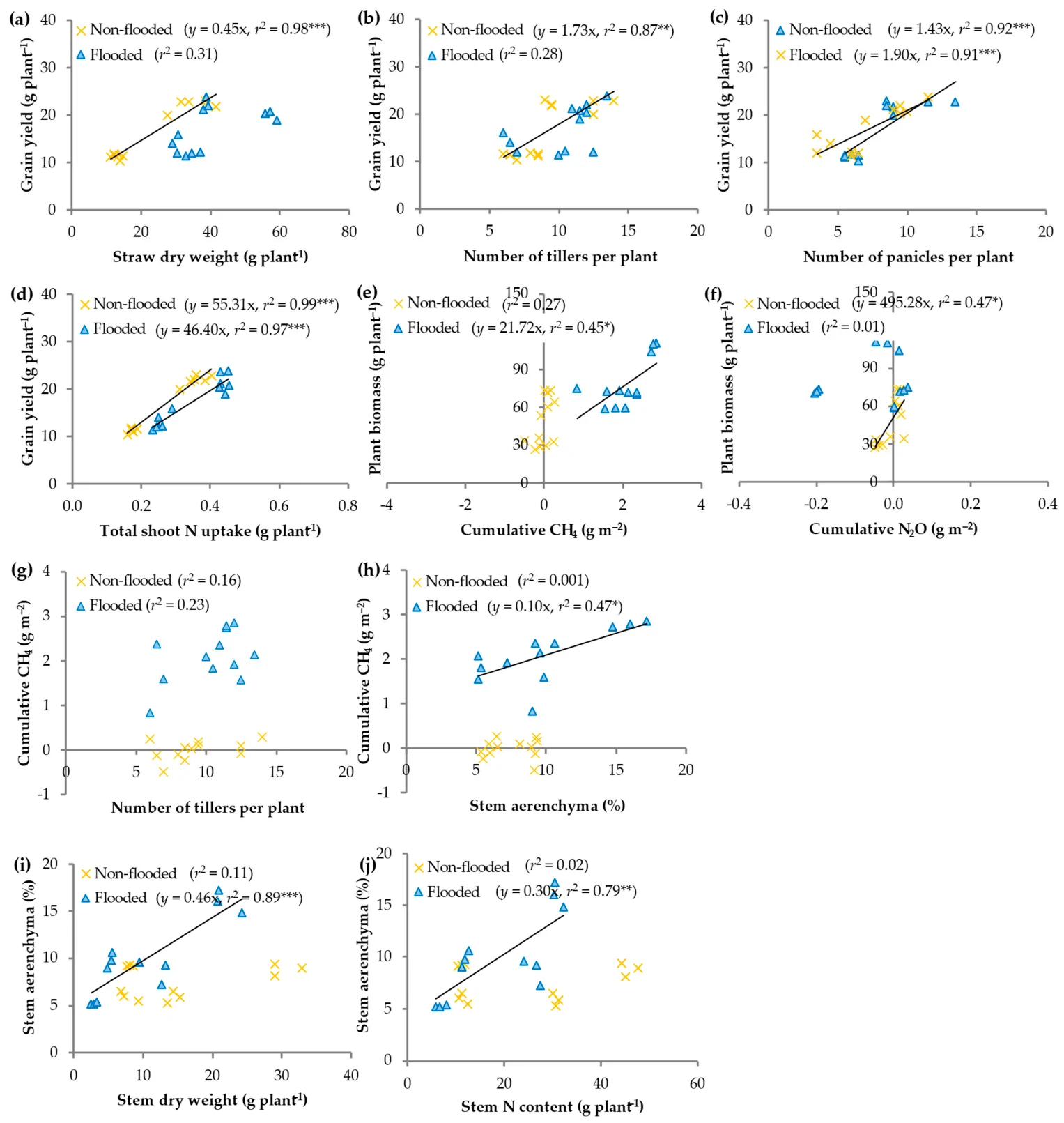
The relationships between grain yield and yield components (**a**–**d**), plant biomass, methane (CH_4_), and nitrous oxide (N_2_O) cumulative emissions (**e**,**f**), morphological traits and CH_4_ cumulative emissions (**g**,**h**), and among stem aerenchyma traits (**i**,**j**). Data are from three independent biological replicates between PTT1 and KDML105 rice varieties under non-flooded and flooded conditions with non-nitrogen and nitrogen fertilizer regimes (*n* = 12). The solid lines are the fitted linear regression lines for each trait. * *p* < 0.05, ** *p* < 0.01, and *** *p* < 0.001.

There were significant positive relationships between the grain yield and yield components under contrasting water and N conditions (Figure 6a–d). Under non-flooded conditions, grain yield was linearly correlated with straw dry weight (*r*^2^ = 0.98, *p* < 0.001), tiller number per plant (*r*^2^ = 0.87, *p* < 0.01), and panicle number per plant (*r*^2^ = 0.52, *p* < 0.001). Under flooded conditions, however, only the number of panicles per plant retained a significant positive correlation with grain yield (*r*^2^ = 0.91, *p* < 0.001). Regardless of water conditions, grain yield was strongly positively correlated with total shoot N content under both non-flooded (*r*^2^ = 0.99, *p* < 0.001) and flooded conditions (*r*^2^ = 0.97, *p* < 0.001). Regarding GHG emissions, plant biomass was significantly correlated with cumulative CH_4_ emissions under flooded conditions (*r*^2^ = 0.45, *p* < 0.05) (Figure 6e), while plant biomass was correlated with cumulative N_2_O emissions under non-flooded conditions (*r*^2^ = 0.47, *p* < 0.05) (Figure 6f). Cumulative CH_4_ emissions were strongly correlated with stem aerenchyma formation under flooded conditions (*r*^2^ = 0.45, *p* < 0.05), except under non-flooded conditions, whereas tiller number per plant showed no significant relationship with cumulative CH_4_ emissions under either condition (Figure 6g,h). Stem aerenchyma formation was further associated with key stem morphological and physiological characteristics under flooded conditions, exhibiting significant positive correlations with stem dry weight (*r*^2^ = 0.93, *p* < 0.001) and N content (*r*^2^ = 0.87, *p* < 0.01) (Figure 6i,j), while these relationships were absent under non-flooded conditions.

## 4. Discussion

### 4.1. Yield, Yield Components, N Uptake, and NUE Between Rice Varieties Grown Under Non-Flooded and Flooded Conditions

This study compared GHG emissions of rice varieties grown under contrasting water and N conditions, using PTT1 and KDML105. PTT1 produced higher grain yield than KDML105 across both water and N conditions, primarily due to its superior capacity for panicle development (Figure 1). This genotypic divergence in N utilization has been mechanistically linked to plasticity in the effective panicle number per plant and the sink size, as reflected by the number of spikelets per panicle [27]. In addition, the total shoot N content and relative RNUE did not differ significantly between varieties. PTT1 demonstrated higher PNUE and ANUE than KDML105 (Figure 2). This suggests that the high-yielding variety possessed enhanced N translocation capacity, greater dry matter accumulation, and a more favorable canopy structure. These characteristics collectively promote photosynthesis and biomass accumulation, representing key agronomic and physiological advantages under flooded conditions [28]. Under non-flooded conditions, RNUE and ANUE are strongly influenced by the water supply, agronomic practices, and microbial activity [29]. Consistent with this, KDML105 exhibited higher RNUE and ANUE under non-flooded conditions than under flooded conditions, suggesting that water conditions play a critical role in determining N utilization capacity in traditional rice. In this study, soil moisture in the sandy-loam field was maintained at field capacity, ranging from 20% to 30% [22]. This moisture range is considered optimal for water and nutrient absorption by plants, as uptake efficiency under such conditions is largely determined by the soil texture, structure, and organic matter (OM) content [30]. These differences in NUE between varieties are reflected in their contrasting root responses under different water and N conditions. Flooded conditions promote root morphological development and enhanced NH_4_^+^ assimilation enzyme activity, whereas other conditions, such as alternating wet and dry regimes, improve the structure, oxidation activity, and C and N metabolism in the roots [31]. Furthermore, high-yielding varieties generally demonstrate greater yield stability and N utilization potential than traditional varieties due to a combination of higher canopy photosynthetic rate, more efficient N allocation, and upregulated expression of N transport-related genes in the roots [32]. Our previous results revealed differential root responses between PTT1 and KDML105 under flooded conditions, with PTT1 demonstrating superior root structural traits. However, the present findings collectively suggest that further investigation of root morpho-physiological traits and aerenchyma among rice varieties under contrasting water and N conditions would provide valuable insight into the mechanisms underlying differential N utilization among varieties.

### 4.2. GHG Emissions Between Rice Varieties Grown Under Non-Flooded and Flooded Conditions

Rice cultivation under flooded conditions creates a severe physiological challenge for plants by filling soil pore spaces with water and, in some cases, submerging the shoots. Since gas diffusion in water is approximately 10,000 times slower than in air, this factor significantly retards atmospheric gas exchange. Residual O_2_ in the soil is rapidly consumed by the roots and microorganisms, creating an anoxic or hypoxic rhizosphere [4]. Under these anaerobic conditions, OM undergoes degradation through four sequential stages, hydrolysis, acidogenesis, acetogenesis, and methanogenesis, ultimately producing CH_4_ as a major by-product [33]. This process can be initiated within one day of flooding and increases steadily throughout the cultivation period [34]. This study employed sandy loam soil with a relatively high OM content of 5.13%, above the typical range for such soils, which generally range from below 1% (very low) to 2–4% (average), with values above 5% considered high [35]. The OM content in sandy loam soils can be further increased beyond 3.5% to 7.5% through repeated organic fertilization and successive organic cultivation practices [36]. For example, CH_4_ fluxes were higher at approximately 90 days after transplantation, whereas N_2_O fluxes remained nearly zero throughout the harvest period [37]. Although this study recorded excessive soil P and K contents, these did not significantly affect GHG emissions; however, they contribute to increased biomass and improved nutrition in rice [38]. In addition, pot or chamber size does not limit gas emissions, but they typically require a minimum inside diameter of 15.2 cm [39].

In this study, GHG emissions under all growing conditions were measured at three critical growth stages in both rice varieties, as previously reported [40]. Cumulative CH_4_ emissions from plants and soil systems were primarily influenced by water conditions and N application (Figure 3). However, cumulative emissions were estimated from only three measurements, which is a limitation of this study. More frequent measurements throughout the entire growing season would provide more accurate estimates of cumulative GHG emissions. A previous study by Saenjan et al. [41] revealed that KDML105 emitted more cumulative CH_4_ than CNT1 (modern improved variety). Our results found that PTT1 and KDML105 had numerically different emissions patterns, but cumulative CH_4_ emissions from the plants and soil were influenced only by water condition and the N regime, with no significant effect of rice variety (Figure 3). This suggests that CH_4_ emissions were primarily driven by anaerobic soil conditions and N fertilizer application, both of which are well-established stimulants of CH_4_ production in rice paddies. Similarly, PTT1 showed a greater increase in total CH_4_ emissions than CNT1 and KDML105, but this difference was not under N fertilizer application. In addition, mid-season drainage and alternate wet and dry conditions can mitigate CH_4_ emissions compared with continuous flooding [19]. N_2_O emissions increased the most sharply at the flowering stage across the rice varieties and treatments, particularly in PTT1 under both water conditions and N regimes (Figure 3). However, N_2_O absorption flux was observed in PTT1 under flooded conditions with N fertilizer application at 26 days after transplanting. This pattern likely reflects the wide temporal variability of N_2_O emissions, which is governed by the nitrate (NO_3_^−^) reaction, leading to sudden emissions and adsorption under flooded conditions [25,26]. Although the composition of root exudates was not directly measured in this study, previous research suggests that root exudates play a key role in stimulating N_2_O emissions by increasing the abundance of NO_3_^−^ reduction genes and releasing specific compounds, including terpenoids, lipids, L-isoleucine, L-valine, and pyridoxine, particularly at about 70 days after transplanting [42]. Moreover, an excessive N supply has been shown to promote methanogen activity while suppressing methanotrophs, with high N application rates potentially increasing the GWP of combined CH_4_ and N_2_O emissions by up to 78% [7,8]. The higher GWP and GHGI observed in KDML105 may be partly attributed to its longer growth duration, which could prolong anaerobic soil conditions and maintain substrate availability for methanogenic microorganisms over an extended period [34]. In addition, the lower grain yield of KDML105 may have further contributed to its higher GHGI compared with the shorter-duration, modern improved variety (Figure 3). Similar to the cumulative estimates, GWP and GHGI were also calculated based on only three measurements. Measurements across the entire growing season would more clearly reflect the effects of variety and management conditions.

### 4.3. Stem Aerenchyma Between Rice Varieties Grown Under Non-Flooded and Flooded Conditions

O_2_ deprivation severely disrupts root metabolism and function, triggering complex physiological and genetic adaptations for survival [13]. A key adaptive response is an increase in internal aeration, which is essential for maintaining root function in flooded soils [43]. Central to this adaptation is the formation of longitudinal aerenchyma channels that facilitate the diffusion of O_2_ from the aerial shoots to submerged root tips. While the presence of aerenchyma is crucial in roots, it also forms a continuous network through the aerial parts of the plant, serving as the primary pathway for gas exchange between the plant and the atmosphere, with peak function observed at the anthesis stage [44]. Previous studies have demonstrated that both the leaf sheath and root significantly contribute to gas transport and GHG emissions. Therefore, further study should investigate root morphology under hydroponic solution, which provides more accurate measurements than pot experiments. In this study, stem aerenchyma was observed in both rice varieties, developing in the lower internodes from the coleoptilar to the first node above the stem base at the heading stage, with prominent development in the 20–30 mm internodes. Under our permanently flooded conditions (maintained at a 7 cm water depth), this placed the coleoptilar to the first nodes in a submerged or semi-submerged state (Figure 4). This partial submergence was significant, as it restricted the diffusion of the gaseous hormone C_2_H_4_, leading to its accumulation in the submerged stem tissues [16]. However, significant interactions were noted between stem aerenchyma formation and stem dry weight and stem N content only under flooded conditions (Figure 6).

The development of aerenchyma is influenced by a complex interaction between agronomic practices and genetic diversity. Key factors include water conditions, genetic diversity, and fertilizer management [17,45,46]. Aerenchyma can be categorized by its formation process (lysigenous and schizogenous) and its developmental trigger, being either constitutive (present regardless of stress) or inducible (formed in response to environmental cues) [43]. This organ-specific response to N is complex and can appear contradictory. In rice aerenchyma root, the sensitivity to C_2_H_4_ is increased under N-deficiency [17]. Both low N fertilizer rates and water stress have been reported to induce aerenchyma formation in rice roots [45]. In contrast to the root response, stem aerenchyma formation follows a different pattern, as our study and others have indicated that N availability can promote aerenchyma formation through an interplay with C_2_H_4_ biosynthesis. Alterations in N availability and form are known to influence C_2_H_4_ production and sensitivity under both normal and stressed conditions [15,47]. This phenomenon can be ascribed to the action of two key enzymes: 1-aminocyclopropane-1-carboxylic acid (ACC) synthase (ACS) and oxidase (ACO). Typically, ACC is synthesized in the roots, loaded into the xylem, and transported to the shoots, where constitutively present ACO enzymes convert it to C_2_H_4_ in the leaves [48]. A previous study demonstrated the interplay between N availability and C_2_H_4_ in Arabidopsis, in which high NO_3_^−^ (10 mM) upregulated ACS and ACO gene expression, triggering a rapid increase in C_2_H_4_ production [47,49]. This was due to *OsACS1* expression being a key regulator of C_2_H_4_-dependent aerenchyma formation, particularly during C_2_H_4_ production under stagnant conditions [50]. In addition, diffusion of C_2_H_4_ was restricted and then sharply accumulated under flooded conditions that potentiate aerenchyma formation in responsive varieties via a burst of ROS-mediated programmed cell death (PCD) in the cortex [17]. This shoot-specific mechanism is supported by studies demonstrating enhanced stem aerenchyma following application of ethephon (a C_2_H_4_-releasing compound) [16]. However, a previous study in rice revealed interactions between genetic and environmental factors on the aerenchyma of aerial parts, such as the stem [14,16], the leaf sheath [14], and the root [6,51]. The present study found an interaction between genetic and agronomical practices, such as water conditions, and N fertilizer application, which influenced the morphology of stem aerenchyma among rice varieties. KDML105 markedly showed higher stem aerenchyma plasticity in response to N fertilizer under flooded conditions. Nevertheless, rice variety had no effect on CH_4_ emissions. Furthermore, the morphological development of stem aerenchyma among rice varieties was influenced by N availability, which interacts with water conditions to regulate the formation of stem aerenchyma due to increases in stem dry weight and N content under contrasting water and N conditions (Figure 5). Moreover, there was a positive correlation between dry weight and N content of the stem only under flooded conditions, while this relationship did not hold in plants cultivated under non-flooded conditions (Figure 6). This is in accordance with Steffens et al. [16] and Ni et al. [52], who revealed that stem aerenchyma may primarily represent an adaptive response that improves plant performance under flooded conditions by reducing the metabolic cost of maintaining living tissues, although they found correlation trends between stem aerenchyma and CH_4_ emissions under flooded conditions (Figure 6). We suggest that stem aerenchyma formation may be reflected as a secondary consequence of facilitated internal gas transport rather than a direct mechanism regulating GHG flux and cumulative emissions. Further investigation into varietal differences in aerenchyma in leaf sheath, stem, and root tissues and/or the rhizosphere and soil microorganism diversity among rice varieties is needed, as these factors influence GHG production. Understanding these mechanisms would provide crucial insight into the physiological basis of diversity for limiting GHG among rice varieties.

## 5. Conclusions

This study demonstrated that growing rice under contrasting water and N conditions resulted in significant differences in grain yield, cumulative CH_4_ emissions, GWP, and GHGI between rice varieties. Supplying water and N generally increased cumulative GHG emissions; however, these estimates were based on only three measurements in this study. More accurate conclusions would be possible if emissions were measured throughout the entire growing season. Notably, the formation of stem aerenchyma was more developed, particularly under flooded conditions and N fertilizer application. However, differences in aerenchyma distribution did not consistently correspond with differences in CH_4_ and N_2_O emissions among rice varieties. Future studies should investigate aerenchyma formation in different plant organs, including leaves, stems, and roots, together with C_2_H_4_ dynamics and rhizosphere microbial communities. A better understanding of these physiological and microbial interactions may improve the identification of agronomical management and rice varieties with lower environmental impacts and provide valuable information for developing more sustainable rice production systems.

## Figures and Tables

**Table 1:** Average responses of stem morphology between PTT1 and KDML105 rice varieties grown under contrasting water and N regimes.

Water Condition	Rice Variety	N Regime	Number of Nodes	Number of Aerenchyma Cell	Stem Radius (mm)
Non-flooded	PTT1	Non-N	4.0 ± 0.00	26.0 ± 0.00	2.54 ± 0.01
		N fertilizer	4.0 ± 0.00	27.0 ± 0.00	2.68 ± 0.03
	KDML105	Non-N	4.3 ± 0.00	27.6 ± 0.33	2.90 ± 0.03
		N fertilizer	5.3 ± 0.25	26.6 ± 0.33	2.92 ± 0.08
Flooded	PTT1	Non-N	4.0 ± 0.00	28.3 ± 0.67	2.59 ± 0.03
		N fertilizer	4.0 ± 0.00	30.0 ±1.00	3.26 ± 0.04
	KDML105	Non-N	4.3 ± 0.48	30.0 ± 0.00	2.91 ± 0.01
		N fertilizer	4.8 ± 0.00	30.0 ± 0.00	3.71 ± 0.03

Values represent the mean ± SE (*n* = 3).

## Data Availability

Data are contained within this article.

## Abbreviations

The following abbreviations are used in this manuscript:

ACC	1-aminocyclopropane-1-carboxylic Acid
ACS	1-aminocyclopropane-1-carboxylic Acid Synthase
ACO	1-aminocyclopropane-1-carboxylic Acid Oxidase
ANUE	Agronomical Nitrogen Use Efficiency
NH_4_^+^	Ammonium
CO_2_	Carbon Dioxide
C_2_H_4_	Ethylene Hormone
GWP	Global Warming Potential
GHG(s)	Greenhouse Gas(es)
GHGI	Greenhouse Gas Emissions Intensity
CH_4_	Methane
NO_3_^−^	Nitrate
N	Nitrogen
NUE	Nitrogen Use Efficiency
N_2_O	Nitrous Oxide
OM	Organic Matter
O_2_	Oxygen
PNUE	Physiological Nitrogen Use Efficiency
PCD	Programmed Cell Death
ROS	Reactive Oxygen Species
RNUE	Recovery Nitrogen Use Efficiency
